# Reproductive Biology, Pollination Optimization, and Hybrid Verification of *Acacia mangium* × *A. auriculiformis*

**DOI:** 10.3390/plants15203212

**Published:** 2026-10-19

**Authors:** Yating Yang, Zhenning Ding, Simin Huang, Yanxin Fan, Sheng Lu, Bigui Su, Tianran Han, Shiyan Mu, Yu Chen, Guochang Ding

**Affiliations:** 1 College of Forestry, Fujian Agriculture and Forestry University, Fuzhou 350002, China; 12304021001@fafu.edu.cn (Y.Y.);; 2 Key Laboratory for Forest Stress Physiological Ecology and Molecular Biology of Fujian Provincial, Department of Education, College of Forestry, Fujian Agriculture and Forestry University, Fuzhou 350002, China; 3 College of Landscape Architecture and Art, Fujian Agriculture and Forestry University, Fuzhou 350002, China

**Keywords:** *Acacia mangium* × *A. auriculiformis*, long-term pollen storage, liquid pollination, paternity testing

## Abstract

*Acacia mangium* × *A. auriculiformis* (hybrid acacia) combines high productivity with strong ecological adaptability and is therefore an important species for plantation establishment and germplasm innovation in southern China. Yet large-scale seed production has been constrained in Fujian by asynchronous flowering of the two parents, interspecific reproductive barriers, and the low efficiency of artificial pollination. The results show obvious spatial and temporal differences in the selected *A. mangium*, providing a basis for identifying suitable pollination windows. Ultra-low-temperature storage effectively maintained the long-term viability of *A. auriculiformis* pollen, providing a practical approach to alleviate asynchronous flowering between the parental species. Optimization of the liquid-pollination nutrient solution, pollen suspension system, and mentor-pollen proportion resulted in relatively high pod-set rates under field conditions for selected treatments. SLAF-seq generated 230,580 SLAF tags and 221,240 population-level SNPs. Combined evidence from SNP genotype concordance, principal component analysis, population structure, and phylogenetic analysis indicates that ZD15 and ZD16 show the strongest genetic evidence consistent with hybrid origin and are therefore regarded as the putative hybrid progeny with the strongest current genetic support, while M2 × DY5 is the most strongly supported candidate parental combination. These findings provide theoretical and technical support for germplasm innovation in hybrid acacia and for the development of more efficient and precise hybrid breeding strategies in *Acacia*.

## 1. Introduction

Hybrid breeding serves as a core strategy for forest germplasm innovation and genetic improvement, which can integrate excellent agronomic traits from biparental parents and generate heterotic progeny with superior growth and yield performance compared with parental lines [1]. *Acacia* species are widely recognized for their fast growth rate, strong stress tolerance, and high-quality wood, and have been extensively applied in the production of pulpwood, construction timber, and other industrial raw materials [2]. In addition, acacia roots can form symbiotic associations with rhizobia to conduct biological nitrogen fixation, thereby improving soil nitrogen availability, sustaining long-term stand productivity, and supporting ecological restoration [3]. As a superior hybrid taxon, *Acacia mangium* × *A. auriculiformis* exhibits pronounced heterosis [4]. This hybrid outperforms its parents in tree height, diameter at breast height, and wood fiber traits [5] and possesses strong ecological adaptability to barren soil and harsh habitats [6,7,8], making it an ideal afforestation and ecological restoration species for low fertility sites. Moreover, its wood yield [9], fiber quality [10] and rotation cycle are comparable to *eucalyptus* [11]. As an alternative and rotational plantation species, hybrid acacia can stabilize economic output and alleviate soil fertility decline caused by continuous single-species monoculture, showing great application potential in southern Chinese plantation systems.

Early breeding of this hybrid mainly depended on the selection of natural hybrid individuals and elite clones [12]. Existing studies have verified that parental flowering synchronization, orchard spatial configuration and pollen dispersal distance are important factors affecting natural hybrid seed yield [13]. In recent years, artificial hybridization has gradually replaced natural selection as the mainstream method for acacia germplasm innovation, and reciprocal controlled pollination and polyploid breeding have promoted targeted hybrid breeding. Nevertheless, several restrictive bottlenecks still hinder large-scale hybrid seed production, including parental flowering asynchrony, immature long-term pollen preservation technology, low efficiency of traditional pollination methods, and uncertain genetic authenticity of hybrid progeny.

In southeastern Fujian, the flowering periods of *A. mangium* (maternal parent) and *A. auriculiformis* (paternal parent) are staggered by approximately 20 days. Such phenological mismatch makes fresh paternal pollen unavailable for the optimal pollination period of the maternal parent, highlighting the necessity of long-term pollen storage. Additionally, variation in stigma receptivity with time of day and canopy position of *A. mangium* stigma receptivity further narrows the effective pollination window. Traditional spot pollination is laborious, pollen-consuming and inefficient, which limits large-scale seed preparation. Liquid pollination can optimize pollen dispersion and pollination efficiency, while its effectiveness is restricted by suspension osmotic pressure, nutrient composition, pollen concentration and operation methods [14,15,16]. Field pollination is also vulnerable to exogenous pollen contamination, and morphological identification cannot accurately verify hybrid authenticity.

This study explored key technical constraints limiting artificial hybridization of *A. mangium* × *A. auriculiformis*. The specific objectives included analyzing *A. auriculiformis* male gametophyte development, clarifying the optimal pollination window of *A. mangium*, screening long-term pollen storage conditions, optimizing liquid-pollination formulation and process, and verifying hybrid progeny genetic authenticity. The findings can help address the technical bottlenecks of artificial hybrid breeding of acacia, and provide solid theoretical support and technical reference for subsequent germplasm innovation and precise hybrid breeding of *Acacia* species.

## 2. Results

### 2.1. Long-Term Storage Performance of A. auriculiformis Pollen

**Figure 1:**
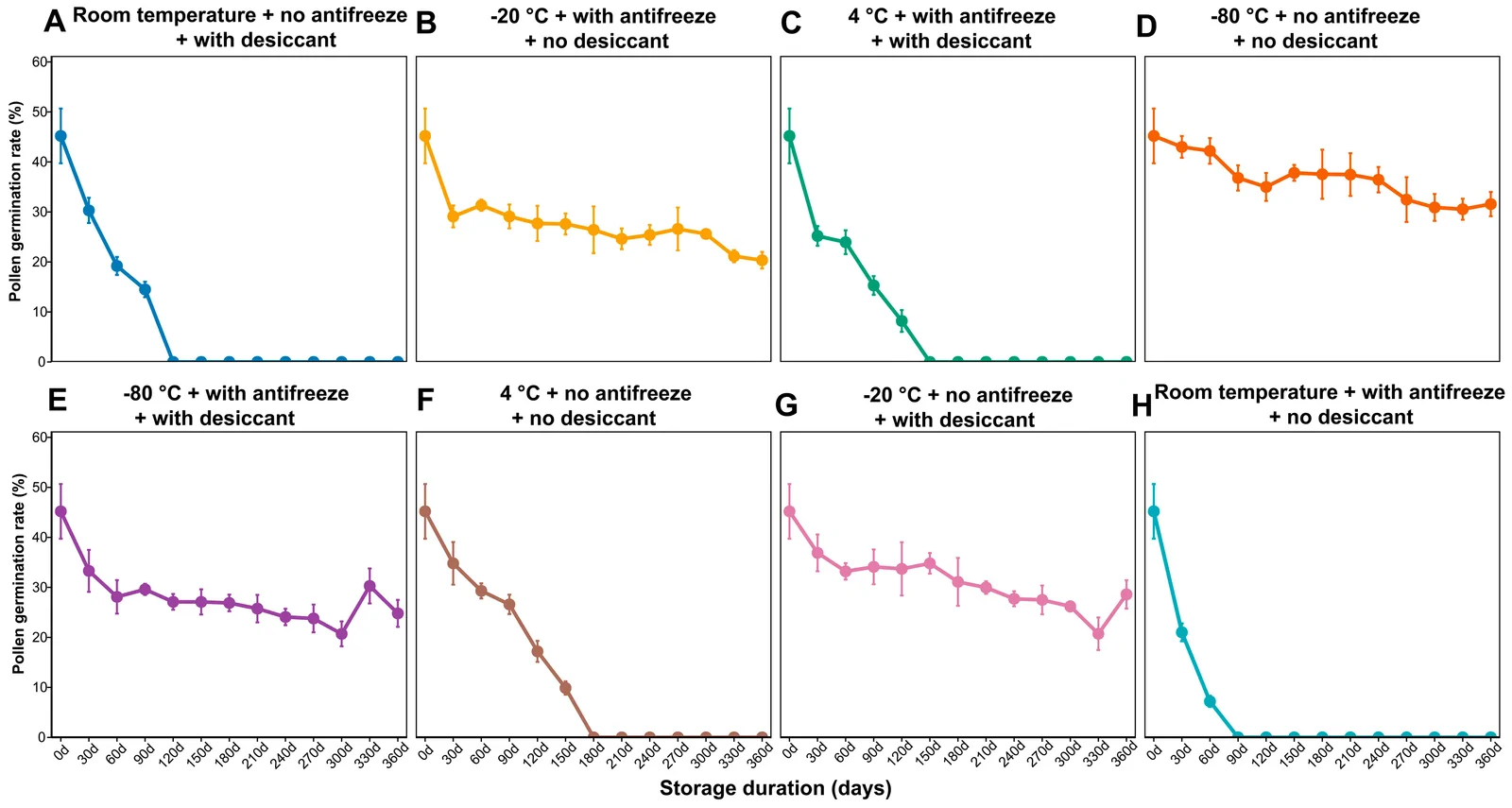
Effects of different storage treatments on the viability of *A. auriculiformis* pollen during 360 days of storage. (**A**–**H**) Changes in pollen germination percentage from 0 to 360 days under different storage treatments. The treatments comprised three factors: storage temperature, cryoprotectant, and desiccant. Pollen viability generally declined with increasing storage duration. After 360 days, storage at −80 °C without anhydrous glycerol or silica-gel desiccant maintained the highest pollen germination percentage and was therefore identified as the optimal long-term storage treatment for *A. auriculiformis* pollen.

Storage temperature, cryoprotectant, and desiccant all affected the long-term storability of *A. auriculiformis* pollen, and their effects varied among treatment combinations. Freshly collected *A. auriculiformis* pollen had an initial germination percentage of 45.22%. Following the eight storage treatments, pollen viability declined progressively with storage duration, although the magnitude of decline differed among treatments (Figure 1). Pollen stored at room temperature or 4 °C lost viability relatively rapidly. Based on the integrated area under the curve (AUC) evaluation (Appendix A), the relative importance of the three factors was storage temperature > cryoprotectant > desiccant. The optimal storage treatment was −80 °C without anhydrous glycerol or silica-gel desiccant. After 360 days of storage under this condition, pollen germination remained at 31.57%, representing a decline of only 13.65% from the initial value. At −20 °C, the treatment with desiccant performed better than the −80 °C treatment containing cryoprotectant.

### 2.2. Pollen Characteristics of A. auriculiformis

**Figure 2:**
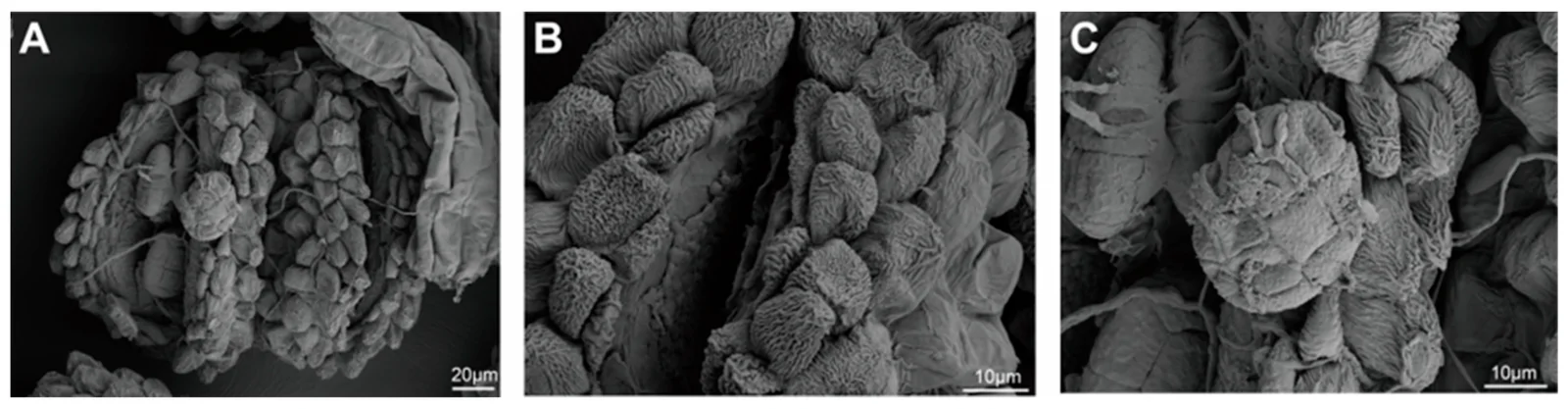
Scanning electron micrographs of *A. auriculiformis* stamens and pollen. (**A**–**C**) Morphological characteristics observed by SEM: (**A**) anther morphology and mode of dehiscence; (**B**) anther dehiscence and internal surface ornamentation; and (**C**) a polyad composed of 16 pollen grains.

Scanning electron microscopy (SEM) revealed the morphological characteristics of *A. auriculiformis* pollen (Figure 2). The stamen consisted of an anther and a filament, with the filament attached to the base of the anther. The anther was generally hemispherical and exhibited conspicuous surface folds and irregular granular protrusions, resulting in relatively coarse surface ornamentation. At maturity, the anther dehisced longitudinally (Figure 2A), with the dehiscence line extending along the longitudinal axis toward both poles, and the anther opened sufficiently to release multiple polyads. The inner surface of the anther was relatively smooth, with granular material adhering to the inner wall (Figure 2B). A single anther locule released multiple polyads (Figure 2A). *A. auriculiformis* produced 16-grained polyads that were subspherical to ellipsoidal in shape (Figure 2C). Each polyad consisted of 16 individual pollen grains arranged in two layers, with eight centrally positioned grains surrounded by eight peripheral grains. The exine ornamentation was regularly arranged and exhibited an approximately reticulate pattern. The exine of the peripheral pollen grains was relatively smooth, whereas groove-like structures were visible in the central region of the inner pollen grains. The mean polar axis length (*P*) and equatorial axis length (*E*) of the polyads were 32.66 ± 1.36 µm and 25.88 ± 1.87 µm, respectively, with a mean P/E ratio of 1.26 ± 0.08. For individual pollen grains, the mean P and E values were 8.27 ± 0.86 µm and 7.07 ± 0.51 µm, respectively, with a mean P/E ratio of 1.17 ± 0.15(Table 1).

### 2.3. Microsporogenesis and Male Gametophyte Development in A. auriculiformis

**Figure 3:**
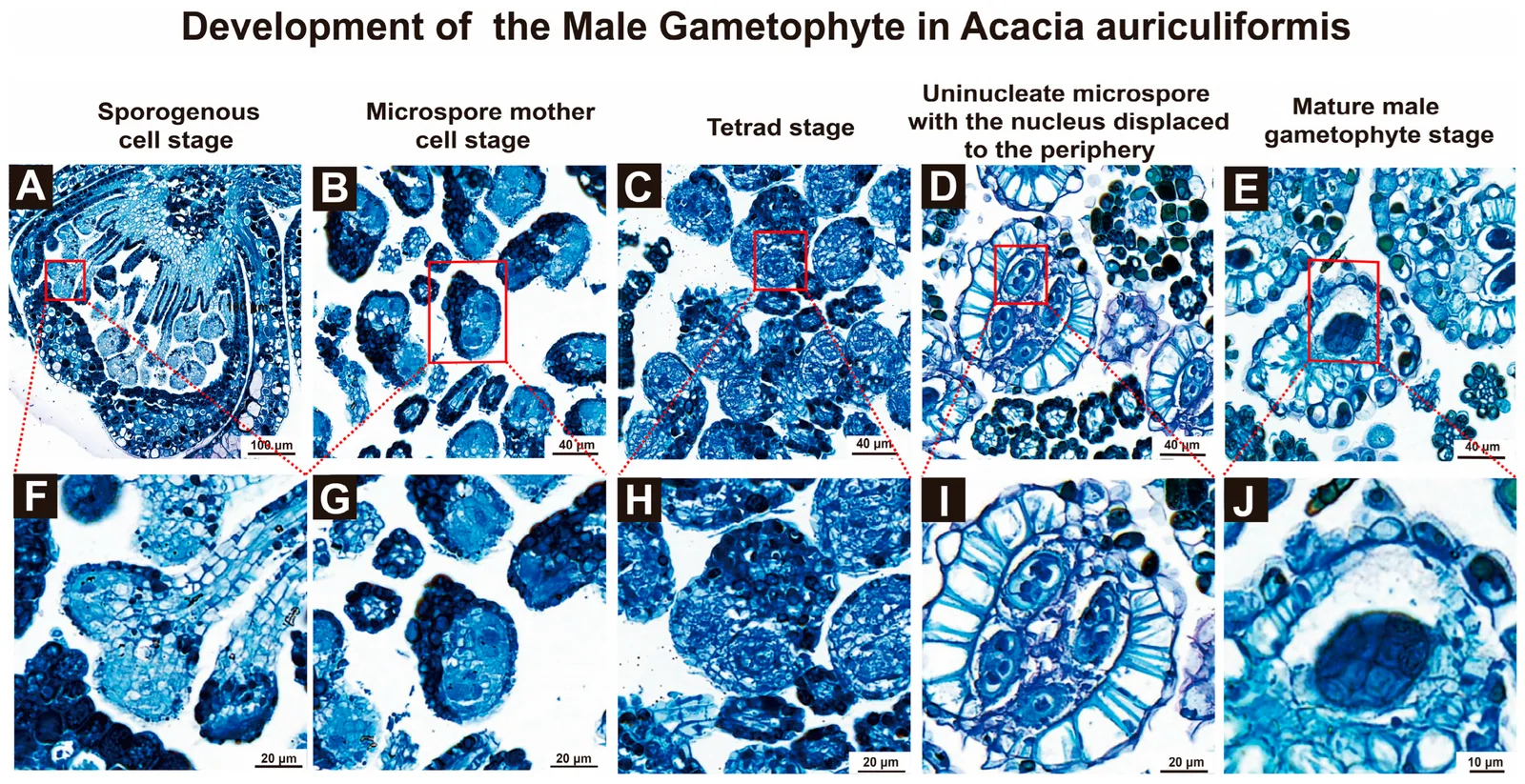
Microsporogenesis and male gametophyte development in *A. auriculiformis*. (**A**–**E**) Microscopic structures at successive stages; (**F**–**J**) enlarged views of the corresponding regions shown in (**A**–**E**). (**A**,**F**) Sporogenous cell stage. (**B**,**G**) M mother cell stage. (**C**,**H**) Tetrad stage. (**D**,**I**) Uninucleate microspore with the nucleus displaced to the periphery. (**E**,**J**) Mature male gametophyte stage.

Paraffin sections of *A. auriculiformis* inflorescences at different developmental stages were examined, and male reproductive development was divided into five stages according to cellular morphology: sporogenous cell stage, microspore mother cell stage, tetrad stage, uninucleate microspore stage with a peripherally displaced nucleus, and mature male gametophyte stage. At the sporogenous cell stage (Figure 3A), large, densely arranged archesporial cells were observed within the anther locules. These cells underwent active mitotic division, resulting in a marked increase in cell number. As development proceeded, the cells enlarged substantially, became nearly spherical or ellipsoidal, and developed increasingly distinct nuclei. During meiosis, each microspore mother cell underwent two successive meiotic divisions to produce four haploid microspores enclosed by a callose wall, forming a tetrad (Figure 3C). After their formation, the microspores remained tightly associated in the tetrad configuration. The microspores subsequently enlarged, with dense cytoplasm and a prominent centrally located nucleus. A conspicuous vacuole then developed and displaced the nucleus toward the cell periphery, corresponding to the uninucleate peripheral-nucleus stage (Figure 3D). Subsequently, the microspore nucleus underwent an asymmetric mitotic division, producing a larger vegetative cell and a smaller generative cell. As development proceeded, the generative cell detached from the pollen wall and became enclosed within the cytoplasm of the vegetative cell. The male gametophyte then matured, ultimately forming a mature 16-grain polyad composed of 16 tightly aggregated pollen grains (Figure 3E).

### 2.4. Temporal and Spatial Variation in Stigma Receptivity of A. mangium

**Figure 4:**
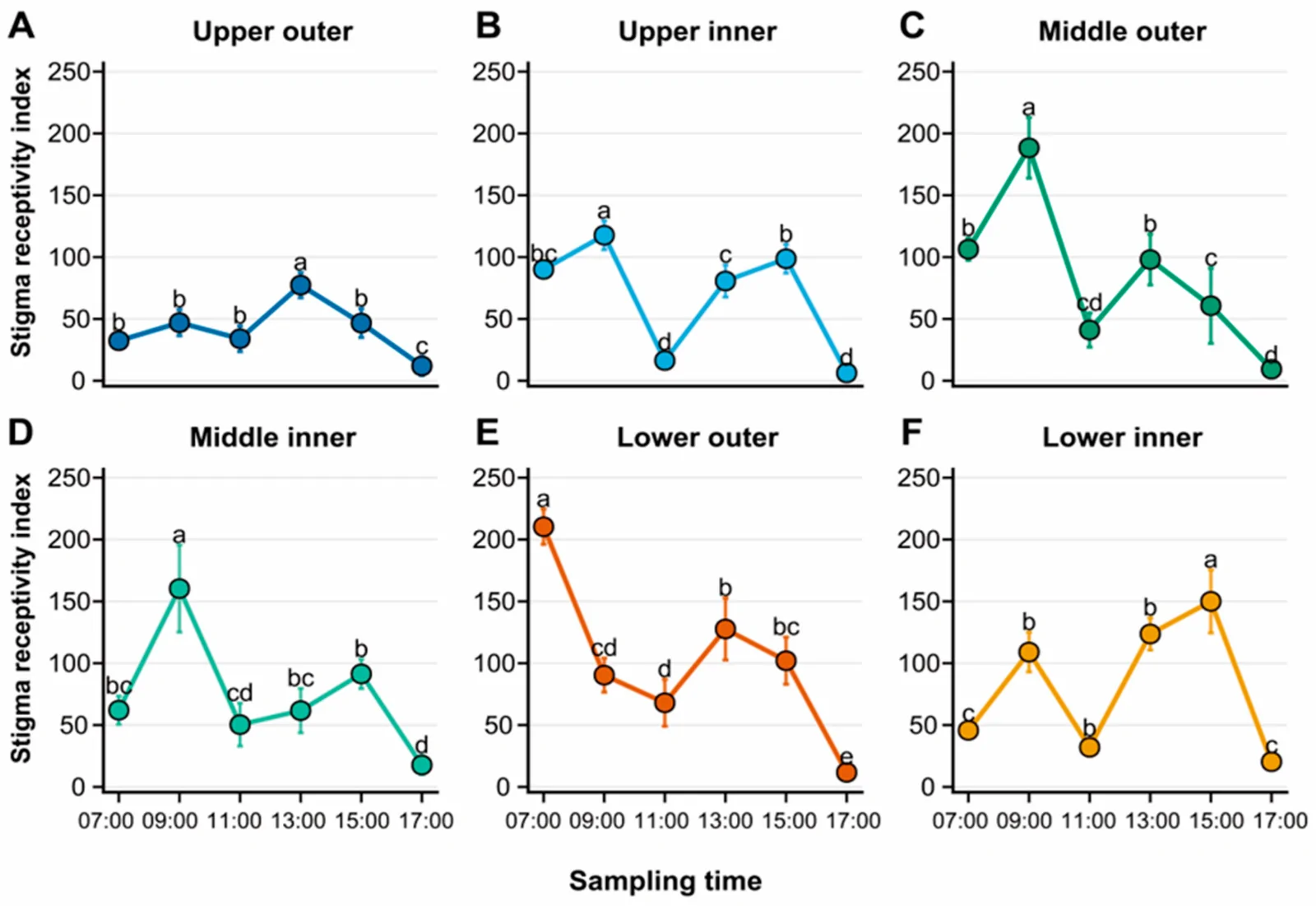
Temporal and spatial variation in stigma receptivity of the maternal parent *A. mangium* during peak flowering. (**A**–**F**) Diurnal patterns of stigma receptivity at six canopy positions. The canopy was divided into upper, middle, and lower layers, each further separated into inner and outer positions, and stigma receptivity was assessed from 07:00 to 17:00. Stigma receptivity generally exhibited an M-shaped diurnal pattern. High receptivity was observed in the outer lower canopy at 07:00 and in both the inner and outer middle canopy at 09:00, indicating suitable pollination windows for controlled hybridization. Sampling time had a significant effect on stigma receptivity at all six canopy positions (one-way ANOVA, all *p* < 0.001). Different lowercase letters indicate significant differences among sampling times within the same canopy position according to Duncan’s multiple range test (*p* < 0.05).

On the selected maternal tree, during peak flowering, stigma receptivity of *A. mangium* varied markedly over the course of the day among canopy layers and between inner and outer canopy positions, exhibiting an overall M-shaped diurnal pattern (Figure 4). At most canopy positions, stigma receptivity reached the first daily peak at 09:00. At this time, stigmas were pale yellow to goose-yellow, with a marked increase in surface bubbles and the appearance of large bubbles, indicating high receptivity. As solar radiation and ambient temperature increased, stigma receptivity declined markedly at 11:00, accompanied by a change in stigma color to yellowish-brown. Subsequently, the stigma receptivity increased again. Stigmas in the outer canopy reached the second peak at 13:00, whereas those in the inner canopy peaked at 15:00. By 17:00, stigmas had become dark brown and receptivity had declined rapidly; few or no bubbles were observed, indicating very weak or nearly absent receptivity.

### 2.5. Optimization of the Liquid Pollination System

#### 2.5.1. Screening of the Liquid Pollination Nutrient Solution

**Figure 5:**
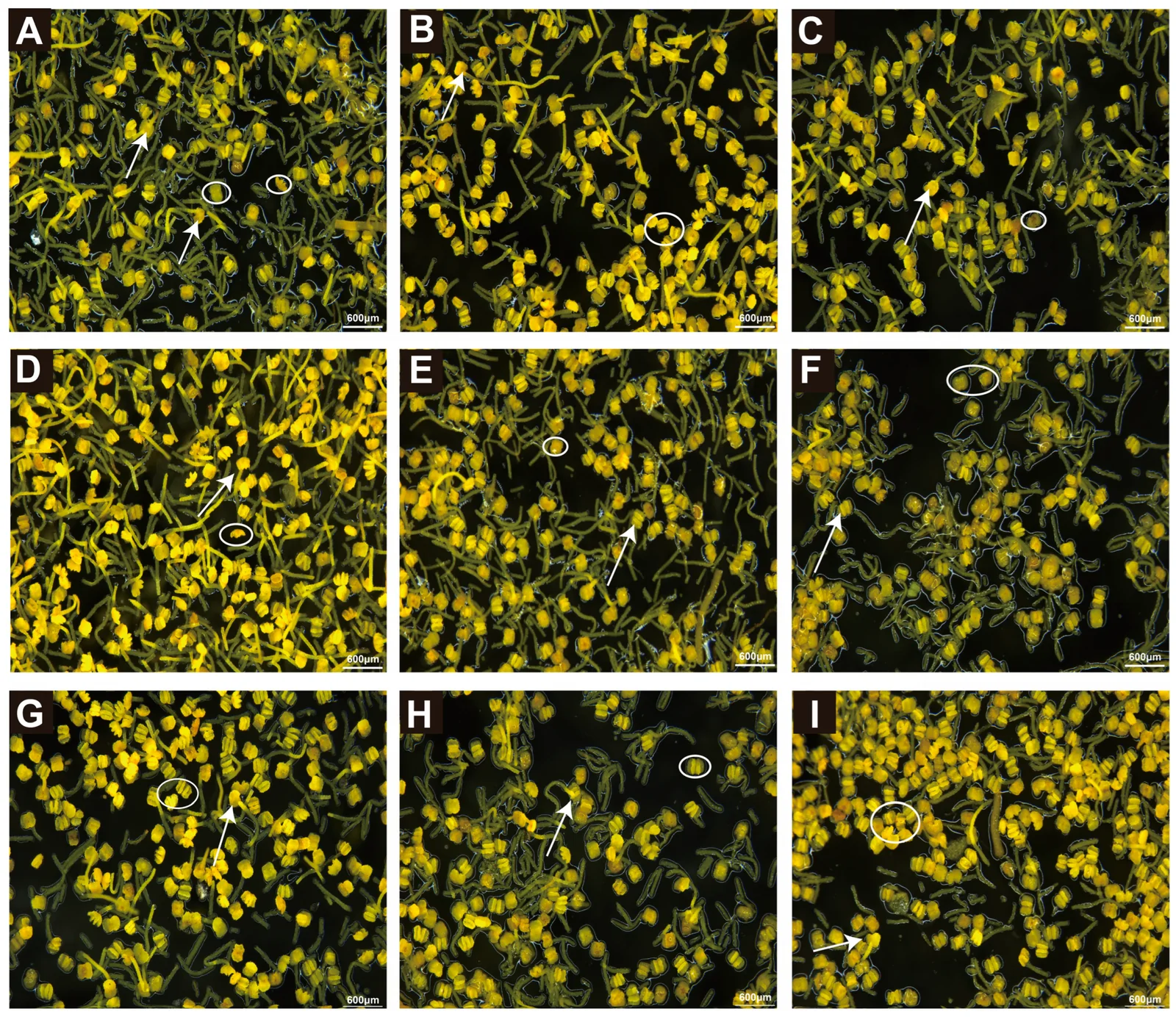
Pollen germination under different pollination-solution formulations. (**A**–**I**) Pollen germination under the nine formulations. Arrows indicate germinated pollen grains, whereas ovals indicate ungerminated pollen grains. Treatment D produced the highest pollen germination percentage and was identified as the optimal formulation: 200 g L^−1^ sucrose + 300 mg L^−1^ boric acid + 10 mg L^−1^ brassinolide at pH 5.0. Suc, sucrose; H_3_BO_3_, boric acid; BR, brassinolide. Scale bar = 600 µm.

On the basal medium containing 200 g L^−1^ sucrose and 300 mg L^−1^ boric acid, both brassinolide (BR) concentration and medium pH affected pollen germination. After 2 h of culture, pollen germination percentages ranged from 23.53% to 40.12%, with Treatment 4 showing the highest value (40.12%). After 4 h, germination percentages ranged from 24.02% to 40.56%, and Treatment 4 again produced the highest value (40.56%) (Table 2). Two-way ANOVA (Table A2) showed that the main effect of BR concentration was not significant (F_2,18_ = 0.136, *p* = 0.874), whereas pH had a highly significant effect on pollen germination rate (F_2,18_ = 5.235, *p* = 0.016). A significant BR × pH interaction was detected (F_4,18_ = 2.939, *p* = 0.049), indicating that the effect of BR on pollen germination depended on medium pH. The overall model was highly significant (F_8,18_ = 2.812, *p* = 0.033) and explained 55.6% of the total variation in pollen germination. Simple-effect analysis (Table A3) showed that under pH 5, 6, and 7 conditions, the simple effects of BR concentration were not significant (all *p* > 0.05). However, at 10 mg L-1, the effect of pH on pollen germination rate was significant (F_2,18_ = 8.480, *p* = 0.003), with the highest germination rate observed at pH 5, which was 40.12%. When BR was 0 and 20 mg L^−1^, the simple effect of pH was not significant (*p* = 0.940 and 0.104). Treatment 4 (200 g L^−1^ sucrose + 300 mg L^−1^ boric acid + 10 mg L^−1^ BR, pH 5.0) therefore represented the optimal nutrient formulation. Because germination under Treatment 4 differed little between 2 and 4 h, a 2 h incubation period was selected to facilitate field application, shorten preparation time, and reduce pollen-tube breakage caused by excessive elongation(Figure 5).

#### 2.5.2. Screening of Suspending Agents

**Figure 6:**
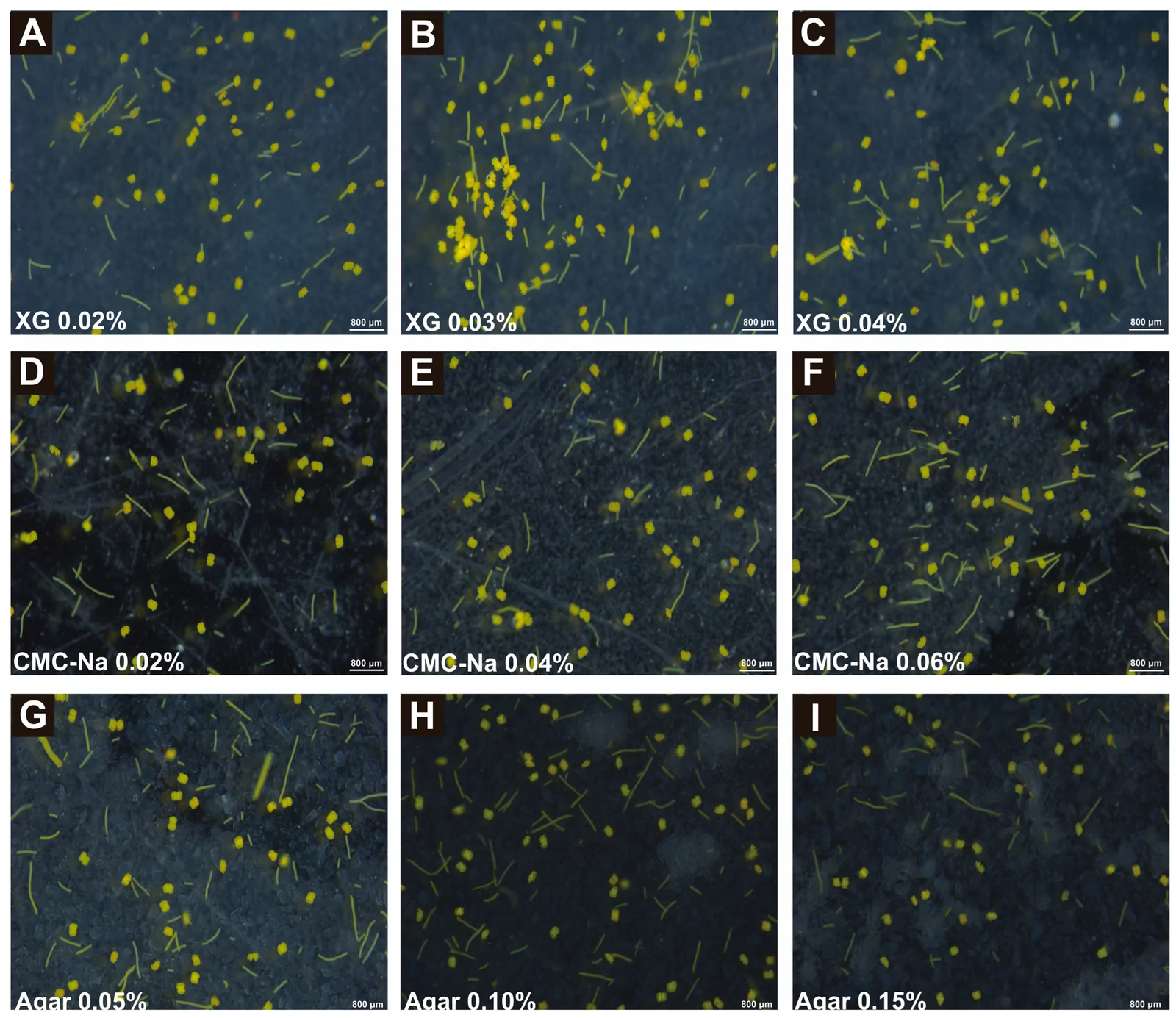
Suspension performance of 0.1% *A. auriculiformis* pollen in different suspending agents and concentrations. (**A**–**I**) Suspension of 0.1% *A. auriculiformis* pollen in nine formulations. Significant differences were detected among suspending agents and concentrations (*p* < 0.05). Treatment B contained the largest number of pollen grains per microscopic field and showed less sedimentation; therefore, 0.03% xanthan gum was identified as the optimal suspending agent. XG, xanthan gum; CMC-Na, sodium carboxymethyl cellulose; Agar, agar. Scale bar = 800 µm.

Suspension performance differed significantly among concentrations of xanthan gum, sodium carboxymethyl cellulose (CMC-Na), and agar (Table 3). At 30× magnification, the number of suspended pollen grains per field ranged from 37.78 to 56. The 0.03% xanthan gum produced the highest number of suspended pollen grains (56 ± 6.67a) and the best suspension performance, whereas 0.15% agar produced the lowest value (37.78 ± 9.23b). There was no significant difference among concentrations within the xanthan gum or CMC-Na treatments (Figure 6). CMC-Na and agar showed relatively poor suspension stability, with pollen readily settling to the bottom and requiring frequent agitation. Therefore, 0.03% xanthan gum was selected as the optimal suspending agent.

#### 2.5.3. Comparison of Pollination Performance Between Stored and Fresh Pollen

**Figure 7:**
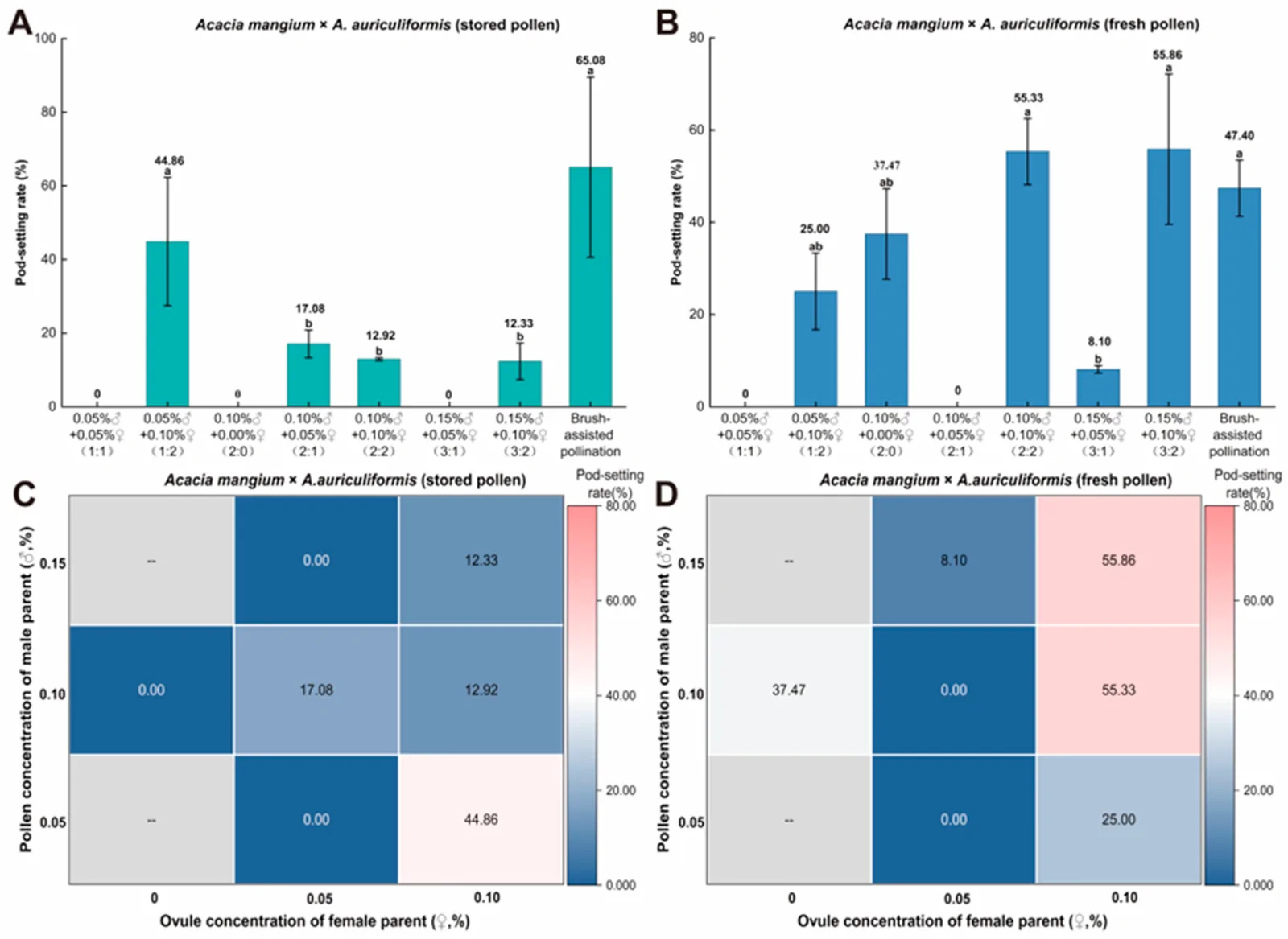
Effects of pollen storage status and concentration combinations on pod set in *A. mangium* × *A. auriculiformis*. (**A**,**B**) Pod-set rates using stored and fresh pollen; (**C**,**D**) heatmaps of pod-set rates under different paternal and inactivated maternal mentor-pollen concentrations. The combination of 0.15% paternal pollen and 0.10% mentor pollen increased pod set 1.48-fold compared with no mentor pollen. Overall, 0.10–0.15% paternal pollen combined with 0.10% mentor pollen produced higher pod-set rates. Different lowercase letters indicate significant differences among treatments based on ANOVA followed by Duncan’s multiple range test (*p* < 0.05).The symbols ♂ and ♀ represent paternal and maternal pollen.

*A. auriculiformis* pollen stored at −80 °C for one year (stored pollen) and freshly collected pollen (fresh pollen) were used to compare pod set between liquid pollination and conventional manual brush pollination (Figure 7). Overall, within the liquid pollination treatments, fresh pollen generally produced higher pod-set rates than stored pollen. Under stored-pollen conditions, pod-set rates differed significantly among pollination treatments (*p* < 0.05). Conventional brush pollination resulted in a pod-set rate of 65.08%, which was higher than that of all liquid pollination treatments. Among the liquid pollination treatments using stored pollen, the combination of 0.05% paternal pollen and 0.10% mentor maternal pollen (1:2) produced the highest pod-set rate, reaching 44.86%. Among the fresh-pollen liquid-pollination treatments, pod-set rates also differed significantly among pollen-ratio treatments (*p* < 0.05). The combinations of 0.15% paternal pollen + 0.10% maternal pollen (3:2) and 0.10% paternal pollen + 0.10% maternal pollen (2:2) produced relatively high pod-set rates of 55.86% and 55.33%, respectively, both of which were significantly higher than those of the other liquid pollination treatments. Overall, pollen freshness, paternal pollen concentration, and the proportion of mentor pollen all influenced pod-setting performance. Although conventional brush pollination produced a higher pod-set rate under stored-pollen conditions, liquid pollination also achieved relatively high pod-set rates under specific pollen-ratio combinations. In the field experiment using fresh pollen, the treatment containing 0.15% paternal pollen + 0.10% inactivated maternal pollen produced a pod-set rate 1.48 times that of the corresponding treatment without mentor pollen.

### 2.6. SNP-Based Parentage Analysis of Putative Hybrid Progeny

#### 2.6.1. Population Genetic Structure and Clustering Analysis

**Figure 8:**
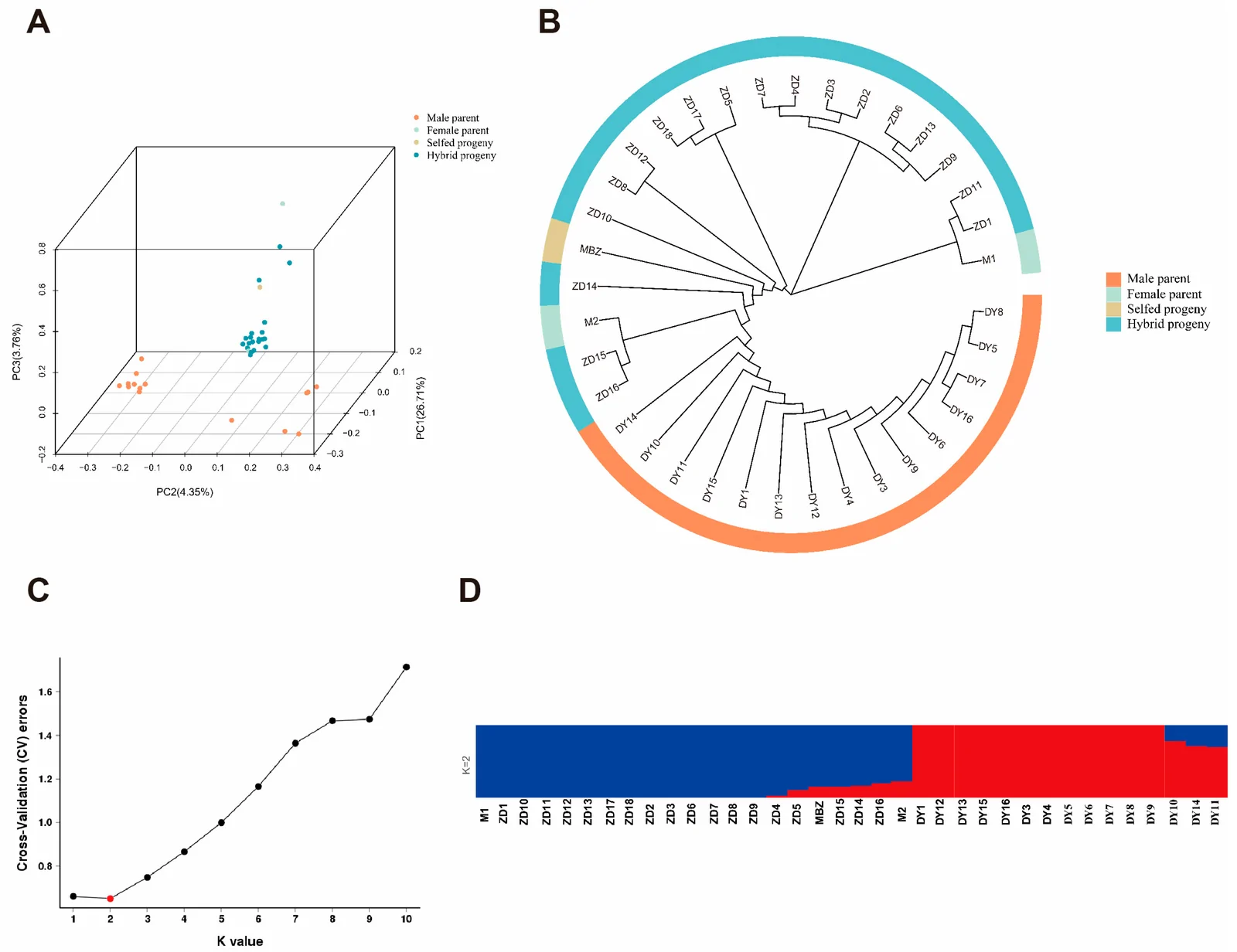
SNP-based analyses of 36 samples comprising parental individuals, putative *A. mangium × A. auriculiformis* hybrid progeny, and one maternal selfed progeny. (**A**) 3D PCA; (**B**) neighbor-joining phylogenetic tree; (**C**) cross-validation error for different K values in ADMIXTURE; and (**D**) population structure at K = 2.Blue represents the maternal parent (*A. mangium*), while red represents the paternal parent (*A. auriculiformis*).

SLAF-seq sequencing of 36 samples generated a total of 230,580 SLAF tags, including 4290 polymorphic tags and 221,240 population-level SNPs. PCA, population structure analysis, and phylogenetic analysis were performed based on the tested materials, which included 15 candidate paternal *A. auriculiformis* individuals (DY1-DY16, excluding DY2), two maternal *A. mangium* individuals (M1 and M2), 18 putative hybrid progenies (ZD1-ZD18), and one maternal selfed progeny (MBZ). PC1, PC2, and PC3 explained 26.71%, 4.35%, and 3.76% of the total genetic variation, respectively, respectively, with a cumulative contribution rate of 34.82% (Figure 8A). *A. mangium* and *A. auriculiformis* were distributed in distinct regions of the PCA space, indicating genetic differentiation between the parental species. Candidate paternal individuals also showed intragroup variation, potentially associated with provenances, families, or genotypes [17]. Putative hybrids generally were located between the parental groups along PC1, while some were closer to the maternal group along PC2, demonstrating a genetic admixture pattern consistent with genetic contributions from both parental species. Population structure analysis (K = 1–10) showed the lowest cross-validation error at K = 2 (Figure 8C). The paternal group was dominated by Q1, whereas the maternal individuals, MBZ, and putative hybrids were dominated by Q2, with varying degrees of admixture. ZD15 comprised 38.35% Q1 and 61.65% Q2, and ZD16 comprised 30.14% Q1 and 69.85% Q2, a pattern consistent with contributions from both parental groups but showing greater similarity to the maternal group, consistent with the PCA results. The neighbor-joining tree separated the samples into two major clades, one dominated by candidate paternal individuals and the other by maternal individuals, MBZ, and putative hybrids (Figure 8B). ZD15 and ZD16 clustered closely with M2 while showing detectable genetic components associated with the paternal group, a pattern consistent with contributions from both parental groups.

#### 2.6.2. Verification of Putative Hybrid Progeny and Paternal Parentage

Paternity of the 18 putative hybrids was evaluated using 30 candidate parental combinations comprising two maternal *A. mangium* individuals and 15 paternal *A. auriculiformis* individuals. SLAF-seq identified 4868 core SNP loci, of which more than 3647 were successfully genotyped in each progeny (Table 4). Parentage consistency was assessed using the abnormal-locus ratio. Among the candidate combinations, M2 × DY5 showed the highest overall genetic concordance with ZD15 and ZD16, which had abnormal-locus ratios of 2.39% and 2.93%, respectively. Combined with the PCA, population structure, and phylogenetic results, M2 × DY5 received the strongest genetic support among the tested parental combinations, while ZD15 and ZD16 showed the strongest evidence consistent with hybrid origin from this parental combination.

## 3. Discussion

### 3.1. Low Temperature Promotes Long-Term Storage of A. auriculiformis Pollen

Pollen viability is fundamental to successful hybridization [18]. In this study, storage temperature was the primary factor affecting long-term viability of *A. auriculiformis* pollen. Storage at −80 °C and −20 °C was more effective than at 4 °C or room temperature, whereas anhydrous glycerol and silica-gel desiccation provided no additional benefit. *Acacia* pollen is sensitive to temperature and humidity, and their interaction affects pollen viability and pollen-tube growth. Pollen water status may therefore interact with temperature to determine storability [19]. The positive effect of low-temperature storage observed here agrees with findings for coconut [20], ash [21], European hazelnut [22], and Prunus sibirica [23]. Low temperature may prolong pollen viability by suppressing metabolic activity and reducing ROS-induced oxidative damage [24]. However, successful cryostorage also depends on pollen water content and freezing–thawing conditions, because intracellular ice formation can damage membranes and organelles [25]. Notably, the lower AUC-based performance of the silica-gel treatment compared with the non-desiccated treatment suggests that long-term storage of *A. auriculiformis* pollen may depend on maintaining an appropriate moisture status rather than maximizing dehydration. Likewise, the addition of anhydrous glycerol as a cryoprotectant reduced pollen viability, possibly because direct contact with glycerol altered pollen water relations and osmotic balance. As the initial pollen moisture content and the osmotic effects of glycerol were not directly assessed, these mechanisms require further validation through measurements of pollen moisture content, membrane integrity, and osmotic regulation. Oxidative stress may also contribute to viability loss during storage; however, ROS accumulation and antioxidant responses were not quantified in the present study. Future work should therefore integrate ROS and antioxidant measurements under defined storage conditions to further elucidate the physiological mechanisms underlying long-term pollen viability loss.

### 3.2. Optimized Liquid Pollination and Mentor Pollen Improve A. mangium × A. auriculiformis Hybridization

Optimization of the liquid pollination formulation, selection of an appropriate pollination window, and use of mentor pollen contributed to improved pod-setting performance in pollination treatments involving *A. mangium* × *A. auriculiformis*. *Acacia* species bear bisexual flowers in heads or spikes, making conventional emasculation of individual flowers impractical; consequently, controlled pollination is commonly conducted at the inflorescence level [4]. Previous methods have included direct contact with intact paternal inflorescences of *A. auriculiformis* [26], pollen collection in polyethylene tubes followed by insertion of the maternal inflorescence [27], and spray or brush pollination [28]. *A. mangium* bears densely arranged bisexual flowers in spikes, with approximately 172 flowers per inflorescence and 83 stamens surrounding each pistil [29]. Natural pollen transfer among inflorescences is also facilitated by electrostatically charged bees [30,31], supporting the use of whole inflorescences as practical pollination units. *A. auriculiformis* produces 16-grained polyads with surface ornamentation that may enhance contact and adhesion to stigmas and pollinators. Its male reproductive development progressed through the sporogenous cell, microspore mother cell, tetrad, uninucleate peripheral-nucleus, and mature male gametophyte stages. The glandular tapetum supplies nutrients, enzymes, and sporopollenin precursors during microspore development and pollen-wall formation [32], and abnormal timing of tapetal degeneration can impair male gametophyte development and cause male sterility [33]. Although the present study characterized male gametophyte development based mainly on cytological morphology, nuclear DNA content was not directly examined. Future studies could determine the DNA content or ploidy of vegetative and generative nuclei to screen for potential ploidy abnormalities during male gametophyte development and to provide additional information on nuclear stability.

Liquid pollination can improve operational efficiency and reduce pollen use [34], making it suitable for large-scale *Acacia* hybridization. A medium containing 200 g L^−1^ sucrose and 300 mg L^−1^ boric acid, previously shown to promote *A. auriculiformis* pollen germination [35], was used as the basal formulation. Brassinolide (BR), a polyhydroxylated steroidal plant growth regulator [36], can promote pollen germination and pollen-tube elongation [37]. Similar concentration-dependent responses have been reported in Dianthus [38] and sweet cherry [39]. Medium pH is also important because pollen-tube tip growth depends on proton gradients, Ca^2+^ dynamics, and cell-wall-associated enzymes [40], and optimal pH differs among species [41,42,43,44]. Here, BR and pH interacted significantly: 10 mg L^−1^ BR enhanced pollen germination at pH 5.0, whereas germination declined under neutral to alkaline conditions. Thus, a mildly acidic medium may favor the positive response of *A. auriculiformis* pollen to exogenous BR.

The diurnal and spatial variation in stigma receptivity further defined the optimal pollination window. *A. mangium* stigma receptivity showed a bimodal daily pattern. The midday decline was likely associated with increased radiation and temperature and reduced humidity, which may accelerate stigma dehydration and reduce peroxidase activity [45]. High temperature can also suppress antioxidant activity and promote ROS accumulation and pistil injury [46], consistent with observations in *Camellia oleifera* [47]. Receptivity partially recovered later in the day as radiation declined. The middle and lower canopy may provide a more stable microclimate because of shading by the upper canopy. In *Acacia melanoxylon*, insect visitation is concentrated in the morning [48], coinciding with the morning receptivity peak observed here. This study observed significant temporal and canopy-position variations in stigma receptivity on selected mother trees during peak flowering; inflorescences in the middle and lower parts of the tree exhibited higher receptivity between 7:00 and 9:00 a.m. Based on these intraspecimen variations, this time window and canopy position can serve as priority candidates for artificial pollination, providing guidance for improving the precision of pollination operations. Future studies could include additional independent plants as biological replicates to verify the consistency of these spatiotemporal patterns across different individuals and their representativeness of the population.

Although conventional brush pollination resulted in a higher pod-set rate under stored-pollen conditions, the optimized liquid-pollination treatments still achieved relatively high levels of pod set. Compared with manual pollination of individual inflorescences, liquid pollination enables a larger area to be treated within a shorter period, thereby facilitating concentrated pollination during the period of high stigma receptivity, improving the utilization of the available pollination window, and providing a potential technical basis for future mechanized or UAV-assisted large-scale artificial pollination. Field trials further showed that adding mentor pollen of *A. mangium* to viable *A. auriculiformis* pollen increased pod-set 1.48-fold relative to treatments without mentor pollen, indicating that the addition of mentor pollen was associated with higher pod set. Similar mentor pollen strategies have already been applied to *Populus* [49], *Rosa* [50], and *Lagerstroemia* [51], with related studies indicating their potential to improve reproductive outcomes in interspecific crosses. Therefore, the observed increase in pod-set rate in this study suggests that mentor pollen may improve pod-setting performance in pollination treatments involving *A. mangium* × *A. auriculiformis*. However, this study did not directly examine heterospecific pollen germination, pollen tube growth, or fertilization. Therefore, whether the observed increase in pod set reflects the alleviation of pre-fertilization reproductive barriers or an increase in actual hybridization success remains to be determined. Future studies could combine pollen tube fluorescence observation and fertilization process analysis to further clarify the mechanism of mentor pollen and its effect on actual hybridization success.

### 3.3. SNP-Based Paternal Parentage Assignment Provides Genetic Evidence for Putative Hybrid Progeny

SNP-based parentage analysis is useful for evaluating the genetic origin of putative hybrid progeny when the maternal parent is known, particularly in tree breeding where controlled crossing is labor-intensive and field pollination may involve unintended pollen sources. The breeding-without-breeding framework likewise uses molecular markers to reconstruct pedigrees and recover paternal ancestry [52,53]. For non-model species, SLAF-seq can generate large numbers of SNPs for genetic differentiation and relatedness analyses [54,55,56,57]. For genetic authentication, paternal assignment should rely primarily on Mendelian compatibility and genotype concordance across multiple informative SNP loci, whereas PCA, population structure, and phylogenetic analyses provide complementary evidence. In this study, 4868 core SNPs were retained for parentage assessment. SNP genotype concordance, PCA, population structure, and phylogenetic analysis consistently provided the strongest support for the M2 × DY5 parental combination. Among the evaluated progeny, ZD15 and ZD16 showed the strongest molecular evidence consistent with hybrid origin. Their abnormal-locus ratios were 2.39% and 2.93%, respectively. Although slightly above the 2% reference threshold, these values remained among the lower mismatch levels across candidate parental combinations and were consistent with the other genetic analyses. Thus, the slight exceedance of the reference threshold should not be interpreted in isolation as evidence against their putative hybrid origin; ZD15 and ZD16 are more appropriately regarded as the putative hybrid progeny with the strongest current genetic support.

Because *A. mangium* and *A. auriculiformis* are genetically differentiated, sequence divergence and potential structural variation between the parental genomes may increase alignment and genotyping inconsistencies when no high-quality reference genome is available; however, these mechanisms were not directly tested here. Future work could strengthen genetic authentication by expanding progeny and independently re-genotyping highly informative SNPs or applying higher-depth resequencing. In addition, whole-genome sequencing of hybrid *A. mangium* × *A. auriculiformis* could be undertaken to establish a high-quality reference genome and to characterize parental genomic contributions and potential structural variation. Such a reference would provide a more robust genomic framework for SNP alignment, genotyping, and parentage assignment. Embryological observations could further clarify whether reproductive barriers act before or after fertilization. With larger progeny populations, the validated SNP resource could also support genetic-map construction and subsequent QTL mapping or GWAS of growth and breeding traits, extending the present pedigree analysis toward marker-assisted and precision breeding.

## 4. Materials and Methods

### 4.1. Plant Materials

**Figure 9:**
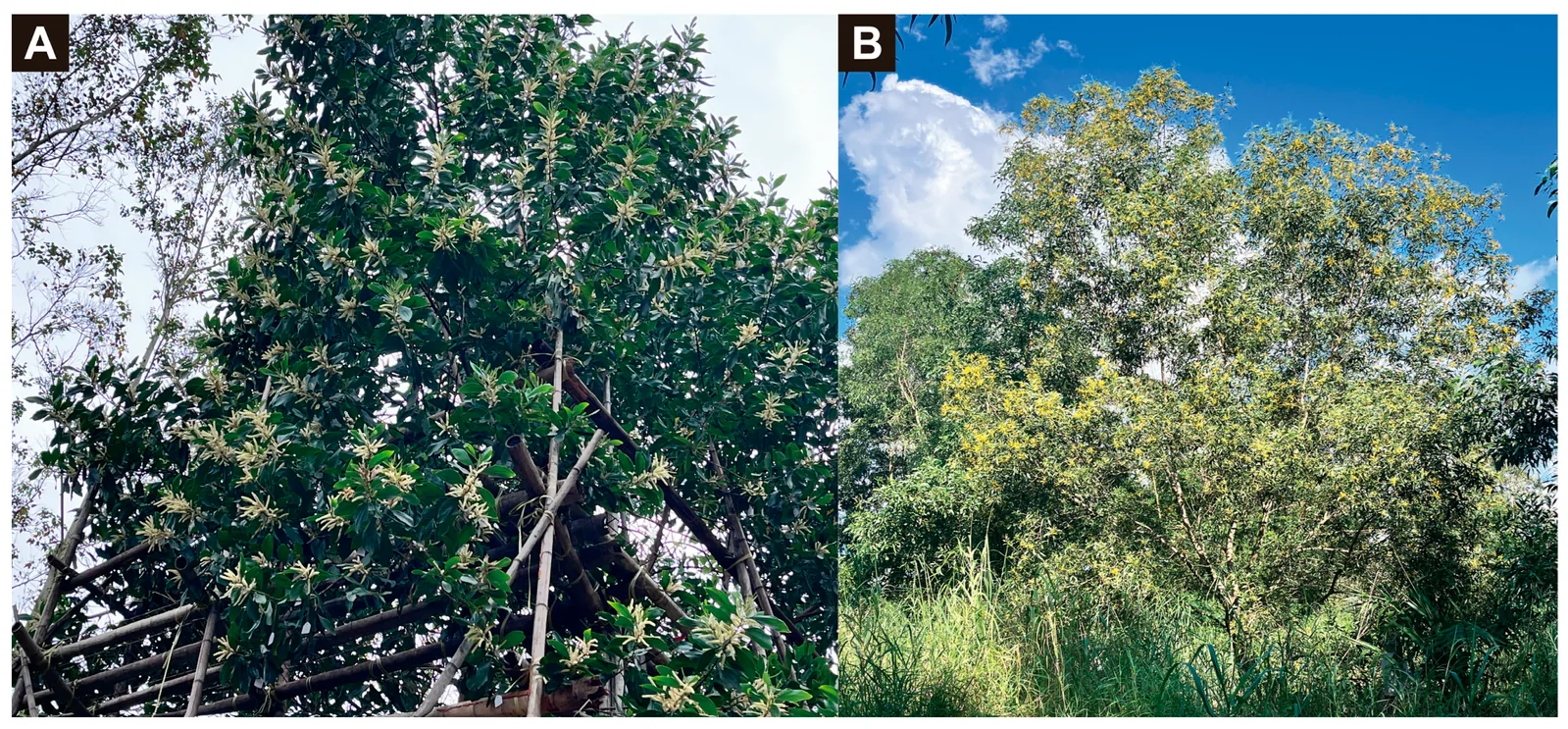
Parental plants used in the study. (**A**) *A. mangium*, the maternal parent; (**B**) *A. auriculiformis*, the paternal parent.

Prior to the experiment, superior individuals of *A. mangium* and *A. auriculiformis* were selected to identify the target parental trees. The maternal parent used for the target cross was selected from a 7a *A. mangium* seed orchard in the Gehou management area of Zhongxi State Forest Farm, Zhangpu County, Fujian Province, China. Pollen was collected in September 2024 from a 26a *A. auriculiformis* seed orchard at the National Key *Acacia* Improved Variety Base in Xinhui District, Jiangmen City, Guangdong Province, China (Figure 9).

### 4.2. Pollen Storage of Acacia auriculiformis

Fresh *A. auriculiformis* pollen was transported to the laboratory and immediately tested for germination. Pollen was evenly distributed on a medium containing 10% sucrose and 1% agar and incubated at 25 °C for 1 h and considered germinated when the pollen tube length was equal to or longer than the diameter of the pollen grain. For each treatment, three independently prepared pollen samples were used, with one microscope slide prepared from each sample, yielding three independent replicates. Three randomly selected, non-overlapping microscopic fields were examined on each slide, with at least 80 pollen grains counted per field. Before statistical analysis, the observations from the three microscopic fields on each slide were averaged to obtain a single value for each independent replicate. Thus, three independent replicate values (*n* = 3).

A mixed-level orthogonal design evaluated storage temperature (room temperature, 4, −20, and −80 °C), cryoprotectant (none or anhydrous glycerol), and desiccant (none or silica gel) (Table 5). Samples at room temperature and 4 °C were stored directly, whereas those at −20 and −80 °C were pre-frozen in liquid nitrogen. For each treatment, three independently prepared pollen samples were used, with one microscope slide prepared from each sample, yielding three independent replicates. Three randomly selected, non-overlapping microscopic fields were examined on each slide, with at least 80 pollen grains counted per field. Before statistical analysis, the observations from the three microscopic fields on each slide were averaged to obtain a single value for each independent replicate. Thus, three independent replicate values (*n* = 3), and pollen germination was assessed every 30 days.

### 4.3. Reproductive Biology Observations

#### 4.3.1. Pollen Characteristics

Fresh *A. auriculiformis* inflorescences were collected at peak flowering, and mature anthers were isolated. Samples were immediately fixed for 2 h in 2.5% glutaraldehyde prepared in 0.1 mol L^−1^ phosphate buffer and rinsed three times with the same buffer for 15 min each. The samples were then post-fixed in 1% osmium tetroxide (OsO_4_) for 1–2 h and rinsed three additional times. Samples were dehydrated through a graded ethanol series of 30%, 50%, 75%, 80%, 95%, and 100%, for 15 min at each concentration, with absolute ethanol applied twice. Following dehydration, samples were subjected to CO_2_ critical-point drying, mounted on conductive adhesive stubs, and sputter-coated. Pollen morphology was observed and photographed using a scanning electron microscope (SEM4000X, CIQTEK, Hefei, China) at 5 kV. Overall pollen morphology, polar view, equatorial view, and exine ornamentation were recorded. Morphologically intact and clearly oriented pollen grains were randomly selected, and polar axis length (P) and equatorial axis length (E) were measured using ImageJ (version 1.54j; National Institutes of Health, Bethesda, MD, USA), followed by calculation of the P/E ratio. At least five pollen grains were measured per biological replicate, with five biological replicates in total.

#### 4.3.2. Microsporogenesis and Male Gametophyte Development in *Acacia auriculiformis*

In late September 2024, *A. auriculiformis* inflorescences at different developmental stages were collected for paraffin-section observations of microsporogenesis and male gametophyte development. Inflorescences were fixed for at least 24 h in 50% FAA solution (50% ethanol: glacial acetic acid: formaldehyde = 18:1:1, *v*/*v*/*v*) and stored at 4 °C until sectioning. After fixation, samples were softened in hydrochloric acid solution (concentrated HCl: distilled water = 1:1, *v*/*v*), followed by dehydration through a graded ethanol series of 60%, 70%, 80%, 95%, and 100%. The dehydrated samples were progressively cleared in 1/3 turpentine oil (TO) + 2/3 absolute ethanol, 1/2 TO + 1/2 absolute ethanol, 2/3 TO + 1/3 absolute ethanol, and pure TO, and were subsequently infiltrated with a turpentine oil–paraffin mixture followed by pure paraffin. Samples were embedded in paraffin and serially sectioned at a thickness of 8–12 µm using a rotary microtome (RM2016, Shanghai Leica Instruments Co., Ltd., Shanghai, China). After deparaffinization and rehydration through a graded ethanol series, sections were stained with hematoxylin for 7 min and eosin for 2 min, dehydrated, cleared, mounted, and observed using an optical microscope (NIKON ECLIPSE E100, Nikon, Japan).

#### 4.3.3. Stigma Receptivity of *Acacia mangium*

As *A. mangium* at the experimental site represents an introduced cultivated population, some variation in flowering phenology was observed among individual trees. To minimize the influence of differences in flowering stage, one mature tree at full flowering was selected for systematic assessment of stigma receptivity across sampling times and canopy positions. The canopy was divided into upper, middle, and lower layers, and each layer was further separated into inner and outer positions, resulting in six canopy positions. Stigma receptivity was assessed at 07:00, 09:00, 11:00, 13:00, 15:00, and 17:00. At each sampling time and canopy position, three within-tree subsampling replicates were established, with five flowers collected per replicate. Stigma receptivity was evaluated using the benzidine–hydrogen peroxide method. Excised stigmas were immersed for 20 min in a benzidine–hydrogen peroxide solution (1% benzidine:3% hydrogen peroxide: distilled water = 4:11:22, *v*/*v*/*v*), after which stigma coloration and bubble production were recorded.

### 4.4. Screening of the In Vitro Pollen Germination Medium and Optimal Suspension

Based on previous results, 200 g L^−1^ sucrose + 300 mg L^−1^ boric acid was used as the basal germination medium because Ca^2+^ supplementation did not significantly affect *A. auriculiformis* pollen germination [28]. A 3 × 3 two-factor completely randomized design was used to optimize the nutrient solution for liquid pollination. Brassinolide (BR, 0, 10, and 20 mg L^−1^), and pH (5.0, 6.0, and 7.0), yielding nine treatment combinations. The pH of each solution was adjusted with 1 mol L^−1^ HCl or 1 mol L^−1^ NaOH and verified using pH indicator paper (Table 6). Pollen was evenly distributed on microscope slides containing the respective treatment media and incubated at 25 °C. Pollen germination was assessed 2 h and 4 h after inoculation. For each treatment, three independently prepared pollen samples were used, with one microscope slide prepared from each sample, yielding three independent replicates. Three randomly selected, non-overlapping microscopic fields were examined on each slide, with at least 80 pollen grains counted per field. Before statistical analysis, the observations from the three microscopic fields on each slide were averaged to obtain a single value for each independent replicate. Thus, three independent replicate values (*n* = 3) were used for subsequent analysis of variance (ANOVA). Pollen grains with pollen tube were considered viable and germinated. After the optimal liquid pollination formulation had been identified, three types of suspending agents were evaluated, each at three concentration levels, giving nine treatments in total. Xanthan gum was tested at 0.02%, 0.03%, and 0.04% (Treatments 1–3); sodium carboxymethyl cellulose (CMC-Na) at 0.02%, 0.04%, and 0.06% (Treatments 4–6); and agar at 0.05%, 0.10%, and 0.15% (Treatments 7–9).

### 4.5. Assisted Pollination Techniques

Before pollination, flowering branches with an appropriate number of inflorescences and suitable floral maturity were selected. For conventional manual brush pollination, pollen from the paternal parent was applied directly to the stigmas of the maternal parent using a brush. Ten flowering branches were selected from the same mature *A. mangium* maternal tree, with each branch serving as a branch-level experimental unit (*n* = 10). Liquid pollination, *A. mangium* pollen was heat-inactivated overnight at 80 °C as mentor pollen. To evaluate the effects of different pollen mixtures on pod-setting performance, mixtures containing different proportions of viable paternal *A. auriculiformis* pollen and mentor pollen were evaluated. Viable paternal *A. auriculiformis* pollen and inactivated maternal pollen were mixed at ratios of 1:1, 1:2, 2:0, 2:1, 2:2, 3:1, and 3:2 in the optimized suspension and prepared immediately before use. In order to eliminate the influence of differences in genetic backgrounds of different maternal parents on treatment comparisons, supplemental pollination was conducted on one mature *A. mangium* maternal tree. For each treatment, five flower branches were selected from different orientations within the same maternal tree, and the number of inflorescences was recorded. When the target inflorescences reached 50% and 80% flowering, a pollen suspension was sprayed onto the target inflorescences using a sprayer for liquid pollination. Each branch was recorded as a treatment unit within the same maternal tree. After pollination, the number of pods per treatment was recorded, and pod-setting performance was evaluated by the ratio of the number of pods formed on each branch to its number of inflorescences. Based on this, the pod results of different pollination treatments were summarized. The resulting offspring were further subjected to molecular markers for paternal kinship identification.

### 4.6. Paternal Parentage Identification of Putative Hybrid Progeny

Collect young leaves from the maternal parent *A. mangium*, the target paternal parent *A. auriculiformis*, and putative hybrid progeny. Genomic DNA was extracted using a modified cetyltrimethylammonium bromide (CTAB) method. DNA integrity was assessed by 1% agarose gel electrophoresis, and DNA concentration and purity were determined using a NanoDrop spectrophotometer (Thermo Fisher Scientific, Waltham, MA, USA). DNA samples meeting the quality requirements were used for SLAF library construction and sequencing.

Genomic DNA was double-digested with Hpy166II and EcoRV, followed by 3′ A-tailing, dual-index adapter ligation, PCR amplification, purification, and recovery of 314–414 bp inserts. Qualified libraries were sequenced as paired-end 150 bp reads on an Illumina platform by Biomarker Technologies Co., Ltd. (Beijing, China).

Because no suitable reference genome was available, high-quality reads were clustered by sequence similarity using LAST (lastal 759). Sequences originating from the same genomic locus were defined as one SLAF tag, and the sequence with the greatest sequencing depth within each SLAF tag was selected as the representative sequence for that locus.

Reads were aligned to the SLAF reference set using BWA (v0.7.10-r789) [58]. V. SNPs jointly detected by GATK (v3.8) and SAMtools (v1.9) were retained. Genetic relationships were assessed by PCA using smartPCA in EIGENSOFT (v6.0), a neighbor-joining tree in MEGA X [59], and population structure analysis using ADMIXTURE (v1.22) [60].

Based on the filtered high-quality SNP loci, genotype consistency between each putative hybrid progeny and the candidate parental pair was evaluated. The analysis was based on whether the progeny genotype contained alleles that were not detected in either parent, with SNP genotypes compared locus by locus among each progeny, the maternal parent, and the paternal parent.

For each SNP locus, if all alleles detected in the progeny could be accounted for by the genotypes of the two candidate parents, the locus was classified as normal. If the progeny carried an allele that was not detected in either parent, the locus was classified as abnormal. If no valid genotype was obtained for the progeny at a given locus, the locus was classified as missing (lack). Subsequently, the numbers of normal, abnormal, and missing SNP loci, as well as the total number of SNP loci included in the analysis, were calculated for each progeny sample. The abnormal-locus ratio was calculated as follows:

$$\mathrm{Abnormal ratio}\left(\%\right)=\frac{N_{\mathrm{abnormal}}}{N_{\mathrm{abnormal}}+N_{\mathrm{normal}}}\times 100$$

where *N*_abnormal_ represents the number of SNP loci at which the offspring genotype was incompatible with the candidate parental genotypes, and *N*_normal_ represents the number of loci at which the offspring genotype was compatible with the candidate parental genotypes.

Abnormal-locus ratio of 2% was used as an empirical reference threshold in the sequencing pipeline. Samples with an abnormal-locus ratio < 2% were considered to show high genetic consistency with the specified parental combination; ratios of 2–5% indicated a possible genetic relationship, whereas ratios >5% indicated a relatively weak genetic relationship. In general, a lower abnormal ratio indicated a higher likelihood that the individual was derived from the candidate parental combination.

## 5. Conclusions

This study focused on *A. mangium* and *A. auriculiformis* and systematically investigated the key aspects of hybrid seed production, including reproductive biology, long-term pollen storage, optimization of liquid pollination formulations, and paternal parentage analysis of putative hybrid progeny. The results indicate that significant temporal and spatial differences in stigma receptivity were observed within a single mature *A. mangium* maternal tree during full flowering, providing a reference for the selection of artificial pollination windows. Ultra-low-temperature storage effectively preserved the viability of *A. auriculiformis* pollen over extended periods, providing a practical option for overcoming asynchronous flowering between the parental species. Further optimization of the liquid pollination nutrient solution, pollen suspension system, and mentor-pollen ratio resulted in higher pod-set rates, indicating that these factors influence post-pollination reproductive performance. SLAF-seq reduced-representation genome sequencing generated a large number of SNP markers. Combined evidence from SNP genotype concordance, PCA, population structure, and phylogenetic analyses indicated that ZD15 and ZD16 were the putative hybrid progeny with the strongest current genetic support, while M2 × DY5 was the most strongly supported candidate parental combination. Overall, this study systematically investigated several key reproductive and technical constraints in *A. mangium* × *A. auriculiformis* hybrid seed production and optimized selected critical steps, providing a theoretical basis and technical reference for subsequent hybrid breeding and the selection of superior hybrid germplasm.

## Figures and Tables

**Table 1:** Morphological parameters of *Acacia auriculiformis* pollen.

Pollen Type	Polar Axis, *P* (µm)	Equatorial Axis, *E* (µm)	P/E
Polyad	32.66 ± 1.36	25.88 ± 1.87	1.26 ± 0.08
Individual pollen grain	8.27 ± 0.86	7.07 ± 0.51	1.17 ± 0.15

**Table 2:** Effects of different pollination-solution formulations and culture duration on pollen germination percentage.

Treatment	2 h (%)	4 h (%)
1	29.52 ± 8.14 bc	31.09 ± 5.03 bc
2	31.06 ± 0.50 bc	34.67 ± 2.96 abc
3	30.13 ± 3.11 bc	32.91 ± 1.71 bc
4	40.12 ± 8.64 a	40.56 ± 8.72 a
5	25.51 ± 2.86 bc	38.21 ± 3.66 ab
6	23.64 ± 4.31 c	31.51 ± 2.34 bc
7	33.31 ± 6.97 b	38.74 ± 1.84 ab
8	23.53 ± 5.46 c	24.02 ± 4.29 d
9	29.96 ± 1.79 bc	29.48 ± 4.44 c

Note: Different letters in the same column indicate significant differences at the *p* < 0.05 level.

**Table 3:** Effects of different suspending agents on pollen suspension performance.

Suspending Agent	Concentration (%)	Suspended Pollen Grains
Xanthan gum	0.02	55 ± 8.72 a
Xanthan gum	0.03	56 ± 6.67 a
Xanthan gum	0.04	55.44 ± 7.99 a
CMC-Na	0.02	40.11 ± 6.74 b
CMC-Na	0.04	40.44 ± 17.24 b
CMC-Na	0.06	39.44 ± 5.60 b
Agar	0.05	48 ± 10.48 ab
Agar	0.10	47.56 ± 3.37 ab
Agar	0.15	37.78 ± 9.23 b

Note: Different letters in the same column indicate significant differences at *p* < 0.05.

**Table 4:** Statistics of SNP genotyping quality for the cross combination ‘M2 × DY 5’.

Sample	Abnormal Ratio	Abnormal	Normal	Lack	Sum
ZD1	3.68%	140	3664	1064	4868
ZD2	4.59%	181	3764	923	4868
ZD3	4.78%	186	3706	976	4868
ZD4	5.35%	213	3770	885	4868
ZD5	5.17%	211	3872	785	4868
ZD6	4.99%	197	3748	923	4868
ZD7	4.62%	177	3651	1040	4868
ZD8	5.18%	205	3752	911	4868
ZD9	5.76%	228	3729	911	4868
ZD10	4.91%	195	3780	893	4868
ZD11	5.39%	216	3795	857	4868
ZD12	4.86%	189	3702	977	4868
ZD13	4.75%	182	3647	1039	4868
ZD14	4.07%	179	4220	469	4868
ZD15	2.39%	96	3914	858	4868
ZD16	2.93%	123	4073	672	4868
ZD17	4.56%	177	3702	989	4868
ZD18	5.31%	210	3745	913	4868

**Table 5:** Storage treatments for *Acacia auriculiformis* pollen.

No.	Temperature	Cryoprotectant	Desiccant
1	Room temperature	Anhydrous glycerol	None
2	Room temperature	None	Silica gel
3	4 °C	Anhydrous glycerol	Silica gel
4	4 °C	None	None
5	−20 °C	Anhydrous glycerol	None
6	−80 °C	Anhydrous glycerol	Silica gel
7	−20 °C	None	Silica gel
8	−80 °C	None	None

**Table 6:** Nutrient-solution treatments for liquid pollination.

Treatment	Sucrose (g L^−1^)	Boric Acid (mg L^−1^)	Brassinolide (mg L^−1^)	pH
1	200	300	0	5.0
2	200	300	0	6.0
3	200	300	0	7.0
4	200	300	10	5.0
5	200	300	10	6.0
6	200	300	10	7.0
7	200	300	20	5.0
8	200	300	20	6.0
9	200	300	20	7.0

## Data Availability

The data supporting the findings of this study are available from the corresponding author upon reasonable request.

## Abbreviations

The following abbreviations are used in this manuscript:

BR	Brassinolide
XG	Xanthan gum
CMC-Na	Sodium carboxymethyl cellulose
CTAB	Cetyltrimethylammonium bromide
BWB	Breeding and without breeding
M	*Acacia mangium*
DY	*Acacia auriculiformis*
ZD	*Acacia mangium* × *A. auriculiformis* hybrid progeny
MBZ	Maternal selfed progeny
SNP	Single nucleotide polymorphism
QTL	Quantitative trait locus
GWAS	Genome-wide association study

## Appendix A

**Table A1:** Range analysis of the orthogonal long-term storage experiment based on the area under the pollen germination curve.

Factor	Level	Mean AUC (%·d)	R (%·d)	Optimal Level	Rank
Temperature	25 °C	972.59	9384.41	−80 °C	1
	4 °C	2420.85			
	−20 °C	9426.45			
	−80 °C	10,356.99			
Temperature Cryoprotectant	None	6625.27	1662.10	None	2
	Anhydrous glycerol	4963.17			
Desiccant	None	6072.00	555.55	None	3
	Silica gel	5516.45			

Note: AUC represents the area under the mean pollen germination percentage curve from 30 to 360 days, calculated using the trapezoidal rule. A larger AUC indicates better maintenance of pollen germination capacity throughout the storage period. The mean AUC for each factor level was calculated as the equally weighted mean of the corresponding treatments. The range (R) was calculated as the difference between the maximum and minimum mean AUC values among levels of the same factor. Based on the integrated AUC evaluation, the relative importance of the factors was temperature > cryoprotectant > desiccant, and the optimal combination was −80 °C without anhydrous glycerol or silica-gel desiccant.

## Appendix B

**Table A2:** Two-way ANOVA for the effects of brassinolide concentration and pH on pollen germination.

Treatment	SS	df	MS	F	P
BR	0.001	2	<0.001	0.136	0.874
pH	0.030	2	0.015	5.235	0.016
BR × pH	0.034	4	0.008	2.939	0.049
Error	0.052	18	0.003	-	-

Note: A 3 × 3 two-factor completely randomized design was used. The overall model was highly significant (F_8,18_ = 2.812, *p* = 0.033, R^2^ = 0.556).

**Table A3:** Simple-effect tests for brassinolide concentration and pH on pollen germination.

*A. Simple effects of BR concentration at different pH levels*					
pH	SS	df	MS	F	P
5	0.017	2	0.009	3.004	0.075
6	0.009	2	0.005	1.585	0.232
7	0.008	2	0.004	1.423	0.267
*B. Simple effects of pH at different BR concentrations*					
BR (mg L^−1^)	SS	df	MS	F	P
0	<0.001	2	<0.001	0.062	0.940
10	0.049	2	0.024	8.480	0.003
20	0.015	2	0.007	2.570	0.104
